# Response of the Intertropical Convergence Zone to Climate Change: Location, Width, and Strength

**DOI:** 10.1007/s40641-018-0110-5

**Published:** 2018-08-09

**Authors:** Michael P. Byrne, Angeline G. Pendergrass, Anita D. Rapp, Kyle R. Wodzicki

**Affiliations:** 1 Space and Atmospheric Physics Group, Imperial College London, London, SW7 2AZ UK; 2 National Center for Atmospheric Research, Boulder, CO USA; 3 Texas A&M University, College Station, TX USA

**Keywords:** Intertropical convergence zone, Tropical precipitation, Atmospheric dynamics, Climate change, Observations, Models, Theory

## Abstract

**Purpose of Review:**

The intertropical convergence zone (ITCZ) is a planetary-scale band of heavy precipitation close to the equator. Here, we consider the response of the ITCZ structure to climate change using observations, simulations, and theory. We focus on the substantial yet underappreciated projected changes in ITCZ width and strength, and highlight an emerging conceptual framework for understanding these changes.

**Recent Findings:**

Satellite observations and reanalysis data show a narrowing and strengthening of precipitation in the ITCZ over recent decades in both the Atlantic and Pacific basins, but little change in ITCZ location. Consistent with observations, coupled climate models predict no robust change in the zonal-mean ITCZ location over the twenty-first century. However, the majority of models project a narrowing of the ITCZ and weakening mean ascent. Interestingly, changes in ITCZ width and strength are strongly anti-correlated across models.

**Summary:**

The ITCZ has narrowed over recent decades yet its location has remained approximately constant. Climate models project further narrowing and a weakening of the average ascent within the ITCZ as the climate continues to warm. Following intense work over the last ten years, the physical mechanisms controlling the ITCZ location are now well understood. The development of complementary theories for ITCZ width and strength is a current research priority. Outstanding challenges include understanding the ITCZ response to past climate changes and over land versus ocean regions, and better constraining all aspects of the ITCZ structure in model projections.

**Electronic Supplementary Material:**

The online version of this article (10.1007/s40641-018-0110-5) contains supplementary material, which is available to authorized users.

## Introduction

**Fig. 1:**
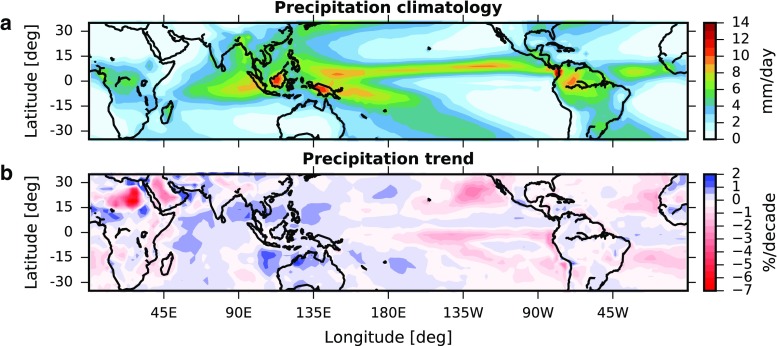
**a** Global Precipitation Climatology Project (GPCP; version 2.3) 2.5° × 2.5° annual-mean precipitation climatology from 1979–2017. **b** Trends in de-seasonalized GPCP monthly-mean precipitation over 1979–2017

Earth’s deep-tropical climate is dominated by the intertropical convergence zone (ITCZ), a narrow band of rising air and intense precipitation (Fig. 1a). Precipitation in the ITCZ is driven by moisture convergence associated with the northerly and southerly trade winds that collide near the equator. The ITCZ accounts for 32% of global precipitation [1] and shapes climate and society in the tropics; any response of the ITCZ to climate change will have implications for tropical regions. It has also been suggested that, through its influence on the global radiation budget, the ITCZ could influence global temperature and precipitation and their responses to climate change [2–5].

The ITCZ moves north and south across the equator following the seasonal cycle of solar insolation, and is intimately connected to seasonal monsoon circulations [6]. In the annual average, the ITCZ lies a few degrees north of the equator [7]. Considerable research has focused on why the ITCZ lies north of the equator, and how this meridional location responds to past and future climate change [8–11]. An energetic theory has been developed over the past two decades to diagnose how processes in Earth’s climate—including radiation asymmetries between hemispheres, atmosphere-ocean coupling, and energy input to the tropical atmosphere—control the zonal-mean ITCZ location [10–21]. This theory has recently been extended to understand variations of the ITCZ location with longitude [22, 23]. Four recent review articles have comprehensively synthesized this body of research on the ITCZ location [1, 24–26]. Here, we provide a broader perspective of the response of the ITCZ to climate change. We focus on two aspects of the ITCZ which have received relatively little attention: its width and strength. In contrast to the ITCZ location, which has not shifted substantially over the past three decades in the Pacific [27] and which shows no robust response in simulations with elevated CO_2_ concentrations [26], the ITCZ width and tropical circulation strength have changed significantly over recent decades [27, 28]. And, the ITCZ width and circulation strength are predicted to continue changing into the future [29, 30]. In climate change simulations, the ITCZ narrows and strengthens in the core of the ascent region as climate warms, a pattern of change which has been termed the “deep-tropics squeeze” [29]. These changes are expected to have important impacts on tropical regions. Our physical understanding of why the ITCZ width and strength change with warming is only beginning to develop [30–38] and represents a key challenge in climate dynamics. Limited understanding of ITCZ width and strength contrasts with our well-developed knowledge of the ITCZ location and the extent of the Hadley circulation [39–42].

Our objectives for this review are to (i) analyze how the ITCZ has changed over recent decades (Observed Changes in the ITCZ), (ii) summarize projections for future changes in ITCZ location, width, and strength from state-of-the-art climate simulations (Projected Changes in the ITCZ Over the Twenty-first Century), and (iii) outline the physical theory underpinning these observed and projected changes (Emerging Theory to Understand ITCZ Responses). We finish with a summary and discussion of priorities for future research (Discussion).

## Observed Changes in the ITCZ

Observational analyses of the ITCZ and its changes rely heavily on satellite-based remote sensing because the majority of the ITCZ lies over oceans where *in situ* observations are sparse. The ITCZ can be readily identified in satellite observations as a region of frequent high clouds with low values of outgoing longwave radiation (OLR), or as a meridional local precipitation maximum (Fig. 1a). A number of studies have automated identification and developed climatologies of ITCZ location using satellite cloud or radiation observations [43, 44] and atmospheric reanalyses [27, 45, 46]. However, many of these earlier studies focused primarily on ITCZ location rather than on ITCZ width or strength.

As satellite cloud and precipitation records have lengthened, these observations have increasingly been used to examine tropical hydroclimate variability and change in response to natural and anthropogenic forcings. Using the first 25 years of the Global Precipitation Climatology Project (GPCP) dataset [47], Smith et al. (2006) [48] showed evidence of temperature-driven precipitation trends in equatorial regions. Lau and Wu [49] examined two blended satellite and *in situ* precipitation datasets and found a shift in the frequency of occurrence of heavy precipitation events from the first to the latter half of the satellite record that was largely driven by increases in heavy precipitation in the ITCZ. An additional study found a band of consistent precipitation increases in the Pacific ITCZ sandwiched between precipitation decreases [50], similar to the pattern shown in Fig. 1b. Using an updated precipitation record, Gu et al. [51] examined interdecadal precipitation variability and again found large precipitation and water vapor increases in the tropical Pacific, with decreases on the margins of the ITCZ. An empirical orthogonal function analysis indicated that most of the changes could be attributed to surface warming and Pacific decadal variability.

Analysis of the 1979–2007 period found negative precipitation trends in the descending branch of the Hadley circulation and positive trends in the ascending branch [52]. To our knowledge, this was one of the first studies to quantify variability in the width of ITCZ precipitation. Considerable regional variability in the ITCZ precipitation width was noted [52]; however, many regions indicated an overall ITCZ narrowing. To further quantify the ITCZ width, Wodzicki and Rapp [27] followed an established methodology [45] to automate monthly identification of the main convergence region associated with the ITCZ center. A series of dynamic and thermodynamic masks were applied to monthly reanalysis data to locate and connect the convergence zones. From the center location, the corresponding observational precipitation record was searched north and south until a minimum precipitation threshold was reached. In this analysis, the north-south distance through the ITCZ center location defines the width of ITCZ precipitation at a given longitude. A 35-year timeseries analysis of ITCZ location, width, and precipitation intensity confirmed and quantified findings by earlier studies [27].

Figure S1 (see Supplementary Material) shows updated 39-year timeseries from 1979–2017 of ITCZ location, width, and precipitation intensity for the Atlantic and Pacific basins using GPCP precipitation estimates and the ERA-Interim reanalysis [53] following the methods of Wodzicki and Rapp [27]. Both the Atlantic and Pacific show no significant trend in the location of the ITCZ (Figure S1a,d). However, this period is characterized by considerable narrowing of the ITCZ in both ocean basins (Figure S1b,e). The estimated ITCZ narrowing corresponds to fractional changes in width of − 20%/K in the Atlantic and − 29%/K in the Pacific, where we have normalized by the change in ERA-Interim global-mean surface-air temperature over 1979–2017. This narrowing accompanies an increase in precipitation intensity (Figure S1c,f); these increasing precipitation trends are especially pronounced in the core of the Pacific ITCZ (not shown). Removal of the large El Niño–Southern Oscillation signal in the first half of the timeseries following the method of Wodzicki and Rapp [27] weakens the significance of the trends, but does not change their qualitative characteristics. A joint analysis of cloud regimes and precipitation suggests that these patterns of precipitation changes can be explained by a shift towards more frequent organized deep convection near the ITCZ center and less frequent convection along its edges [54].

Other observational records of clouds and radiation are consistent with the findings shown in Figure S1. Norris [55] combined surface (1952–1997) and satellite observations (1984–1997) of clouds and radiation and found an increase in upper-level clouds in the central Pacific, with a corresponding decrease in the subtropical regions adjacent to the ITCZ. Concurrent surface-wind observations showed enhancements of convergence and precipitation in the central Pacific. A later study using a longer record of multiple, independent satellite cloud amount and top-of-atmosphere (TOA) albedo estimates found similar differences in cloud patterns between an early period in the satellite observational record (1980s) and a more recent period (2000s) [56]. In the A-Train satellite era beginning in the early 2000s, cloud observations show an increase in opaque, high clouds in the Pacific ITCZ with decreases in surrounding regions [57, 58]. High cloud increases in the Atlantic ITCZ are less pronounced, although reductions in the deepest clouds are present along the ITCZ margins. A recent examination of cloud and radiation datasets also found that tropical-mean high-cloud cover decreases while OLR and precipitation increase with surface warming at interannual timescales [4].

Satellite cloud and precipitation observations tell a coherent story of decreasing ITCZ width and increasing precipitation intensity. These findings from satellite observations are further supported by ocean surface salinity observations. Sea-surface salinity and precipitation minus evaporation (*P* − *E*) are related measures of the hydrological cycle, and there is considerable evidence from ocean observations for a strengthening of the tropical hydrological cycle [59–61]. Long-term trends in salinity show decreases (or freshening) in the Atlantic and Pacific ITCZs but increases along the ITCZ edges [60, 62–64]. The salinity increases in the subtropics and along the ITCZ margins are stronger in the Atlantic, with the freshening more evident in the core of the Pacific ITCZ.

The absence of significant trends in the locations of the Atlantic and Pacific ITCZs over recent decades (Figure S1a,d) might appear to contradict previous studies that showed a southward shift of the ITCZ over the twentieth century associated with large concentrations of scattering sulfate aerosols in the Northern Hemisphere [9, 16]. Aerosol forcing also dried the Asian monsoon region over the same period [65, 66]. However, the rainfall measurements showing a southward ITCZ shift over the twentieth century are from land regions [9, 16]. Because of the short lifetimes of scattering aerosols, their radiative forcing is stronger over land—where the sources are—than over oceans [16]. It is feasible that the ITCZ shifts in different ways over land and ocean because of the contrasting aerosol forcings, which could reconcile the negligible oceanic ITCZ shifts in Figure S1 with the previous studies using land data that found a southward shift. A further consideration is the variation of sulfur emissions over time and between regions. The period shown in Figure S1 (1979–2017) had declining sulfur emissions in North America and Western Europe but periods of increasing and subsequent decreasing emissions in Asia and Eastern Europe [67]. Contrasting trends in emissions and scattering aerosol concentrations in these regions likely affected the ITCZ location in contrasting ways, and could help to explain why we do not observe a clear ITCZ shift over oceans in the last 39 years. Greenhouse gas concentrations were also changing over 1979–2017, and it is well known that the tropical circulation responds differently to aerosol versus greenhouse gas forcings [68, 69]. The relative influences of these forcings on historical trends not only in ITCZ location but also in ITCZ width and strength could be disentangled using single-forcing simulations following Xie et al. (2013) [70], and would be an interesting topic for future work.

## Projected Changes in the ITCZ Over the Twenty-first Century

In this section, we will consider projected changes in ITCZ location, width and strength over the twenty-first century. Numerous studies have examined ITCZ location and its variability but few have focused on its width and strength. As a consequence, metrics for ITCZ width and strength are less established in the literature. Before analyzing projected ITCZ changes, we first define our metrics for ITCZ location, width and strength.

### Metrics of ITCZ Structure

The discussion of observed ITCZ characteristics in the previous section focused on observable quantities, primarily precipitation. However, defining the ITCZ using observations requires choices such as the thresholds of precipitation or OLR used to define the center and edges of the ITCZ. An alternative and intuitive set of metrics for characterizing the ITCZ is based on the atmospheric mass circulation. Characterizing the large-scale tropical circulation directly in observations is not feasible. In models, however, we can apply consistent circulation-based definitions and assess how the ITCZ structure responds to climate change across models. Below we briefly review existing definitions of ITCZ structure before presenting the metrics, based on the mass streamfunction, that we will use to analyze model projections of the ITCZ.

Metrics that have been used to define the ITCZ fall into two broad categories: those based on precipitation and those based on atmospheric mass and energy fluxes. The ITCZ location has been defined as the latitude (i) where the mid-tropospheric atmospheric mass streamfunction is zero [71], (ii) where the poleward flux of moist static energy by the atmosphere vanishes [19], (iii) where tropical precipitation has a maximum [13], and (iv) where the centroid of tropical precipitation lies [17]. Several methods have also been used to define the ITCZ width: (i) the tropical region where there is low-level mass convergence and ascending air on average (the “mass ITCZ” [36]), (ii) the region where there is moisture convergence and *P* − *E* is positive (the “moisture ITCZ” [36]), and (iii) the tropical region with either brightness temperature below a specified threshold (indicative of the presence of high clouds) [72] or rain rate above a specified threshold [27]. The strength of the ITCZ is often left unquantified though obvious metrics include the strength of upward motion averaged over the ITCZ or the maximum zonal-mean precipitation rate. The magnitudes of the location, width and strength of the ITCZ depend on the metrics chosen to quantify them.

To be clear, for this section on model projections, we discuss changes in the ITCZ structure defined in terms of the annual zonal-mean Eulerian-mean meridional streamfunction [73]. The meridional streamfunction, *ψ*(*ϕ*,*p*), is a function of latitude (*ϕ*) and atmospheric pressure (*p*), and quantifies the zonal-mean circulation of atmospheric mass in units of kilograms per second. We choose to focus on the zonal-mean ITCZ as a starting point for understanding the more complex behavior of the zonally varying ITCZ. For example, the well-established energetic theory for ITCZ location was largely developed in a zonal-mean framework [13, 14, 19], and later the mechanistic insights gained from this simplified setup have inspired a more comprehensive theory that takes into account the zonal structure [22, 74]. Our lack of basic understanding of the width and strength of the ITCZ implies that there is still much to learn from the zonal-mean perspective before we proceed to tackling the more complex problem of understanding longitudinal variations.

**Fig. 2:**
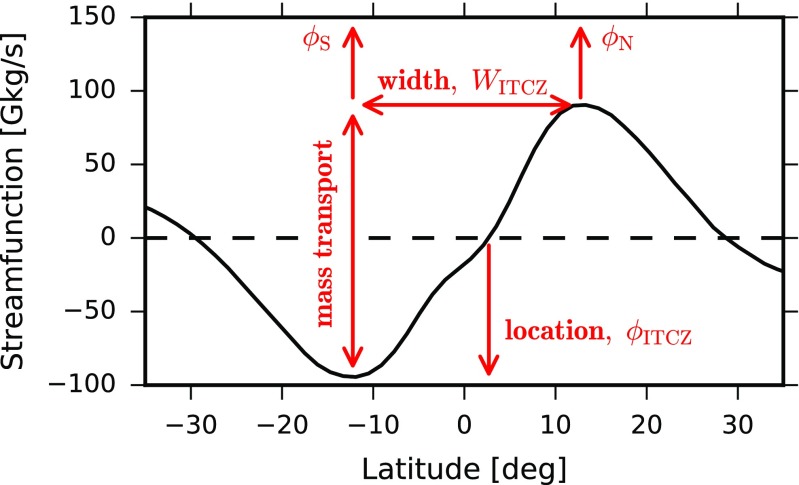
Vertically averaged annual- and zonal-mean meridional streamfunction (700 to 300 hPa with mass weighting) in the historical simulation (1985–2004) for the CNRM-CM5 model. Features of the ITCZ structure are indicated: location (*ϕ*_ITCZ_), width (*W*_ITCZ_), total mass transport (Ψ_ITCZ_), and the northern and southern edges (*ϕ*_N_ and *ϕ*_S_). The ITCZ strength is defined as *ω*_ITCZ_ = −*g*Ψ_ITCZ_/*A*_ITCZ_, where the ITCZ area is given by Eq. 3

The metrics for the ITCZ location, width and total mass transport are indicated on a plot of the mid-tropospheric streamfunction for one climate model (Fig. 2). We define the *ITCZ location* (*ϕ*_ITCZ_) as the latitude closest to the equator where the streamfunction (vertically averaged with mass weighting between 700 and 300 hPa) is zero [71]:

1 $$ \phi_{\text{ITCZ}} = \phi|_{\psi = 0}.  $$

The ITCZ location, defined in this way, represents the latitude of the boundary between the northern and southern Hadley cells. The northern and southern edges of the ITCZ (*ϕ*_N_ and *ϕ*_S_, respectively) are defined as the latitudes closest to the equator at which the meridional derivative of the mid-tropospheric streamfunction is zero, i.e., where *∂ψ*/*∂ϕ* = 0. Equivalently, these are the latitudes at which the time-mean circulation transitions from ascending to descending. The *ITCZ width* (*W*_ITCZ_) is defined as the distance in degrees latitude between these boundaries (Fig. 2):

2 $$ W_{\text{ITCZ}} = \phi_{\mathrm{N}} - \phi_{\mathrm{S}}.  $$

Theories that will be discussed later [36, 37] often focus on ITCZ area rather than ITCZ width, and so for completeness we define the area between the ITCZ edges as the *ITCZ area* [36]:

3 $$ A_{\text{ITCZ}} = 2 \pi a^{2} (\sin \phi_{\mathrm{N}} - \sin \phi_{\mathrm{S}}),  $$

where *a* is Earth’s radius. The total mass transported by the ITCZ (Ψ_ITCZ_) is the difference in streamfunction between the northern and southern ITCZ edges (Fig. 2). We also define a bulk vertical pressure velocity for the ITCZ that is proportional to the total mass transport divided by area:

4 $$ \omega_{\text{ITCZ}} = -g {\Psi}_{\text{ITCZ}} / A_{\text{ITCZ}},  $$

where *g* is the gravitational acceleration. This bulk vertical velocity is defined to be the *ITCZ strength*.

Observational analyses of circulation-based metrics for the ITCZ [(1–4)] are restricted by difficulties in directly observing the large-scale circulation. Consequently, in our analysis of recent ITCZ trends (Figure S1), we use metrics largely derived from satellite observations of precipitation intensity. Climate model trends in ITCZ location [21] and width [30] based on circulation versus precipitation metrics are qualitatively similar, but quantitative comparisons are more challenging. However, for trends in ITCZ strength it is less clear that the circulation and precipitation metrics should scale together as climate changes. Precipitation can be approximated as the product of atmospheric moisture content and circulation strength. The contribution of moisture content to changes in precipitation (the “thermodynamic component”) is well understood [75] but the circulation contribution is not (the “dynamic component”). In our analyses below of projected changes in ITCZ strength we focus on the uncertain dynamic component and use the circulation-based definition (4). We note, however, that a direct comparison between modeled changes in ITCZ strength (defined in terms of circulation) and observed changes (defined in terms of precipitation intensity) is not straightforward given that observed changes in precipitation are due to both changes in circulation and atmospheric moisture content [76].

### Projected ITCZ Responses

Using the circulation-based definitions of the zonal-mean ITCZ location, width and strength outlined above, we calculate projected changes in ITCZ structure over the twenty-first century using 32 state-of-the-art climate models^1^ from the Coupled Model Intercomparison Project Phase 5 (CMIP5) [77]. We examine annual-mean changes between the historical (1985–2004) and RCP8.5 (2079–2098) simulations. A limited number of studies have assessed projected changes in ITCZ location [26], width [30] and strength [29] but these studies have used different sets of simulations and different metrics for the ITCZ. Here, we use a common set of CMIP5 simulations and consistent metrics for ITCZ location, width, and strength to systematically examine projected ITCZ changes. Examining the ITCZ location, width and strength changes side-by-side enables comparison of the robustness in the respective responses.

#### Location

**Fig. 3:**
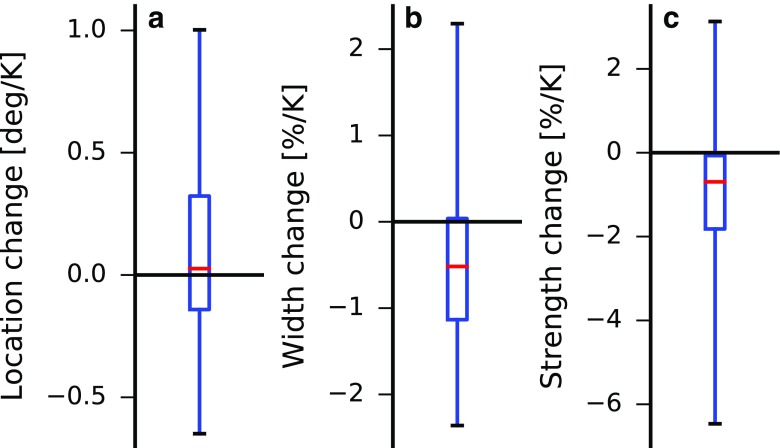
Projected changes in ITCZ **a** location, **b** width, and **c** strength for 32 CMIP5 models between the historical (1985–2004) and RCP8.5 (2079–2098) simulations. The red lines indicate the median model changes, the boxes show the interquartile ranges, and the whiskers show the full model ranges. Note that by the small-angle approximation, sin*ϕ* ≈ *ϕ* close to the equator implying that the fractional changes in ITCZ width shown here are very similar to fractional changes in ITCZ area.

Despite ongoing development of theories for ITCZ location [13–21] and research on its possible migrations over Earth’s history [25, 78–84], few studies have focused on future changes. Our analysis shows that climate models predict no robust change in ITCZ location over the twenty-first century (Fig. 3a). The median model shows a northward shift of 0.03° latitude per kelvin of global-mean surface-air warming, with an interquartile range across models of 0.46°/K. Approximately half of the models analyzed (17/32) predict a northward ITCZ shift with warming, with the remaining models (15/32) predicting a southward shift. The negligible future change predicted by the median climate model is consistent with small changes between pre-industrial control and abrupt 4 ×CO_2_ simulations [26] and a relatively constant annual-mean ITCZ location over recent decades [27] (Figure S1a,d). It has been suggested that interhemispheric radiative forcing asymmetries associated with declining Northern Hemisphere scattering aerosol concentrations over the twenty-first century may drive a northward shift of the ITCZ [16, 85]. If aerosols are indeed driving a northward ITCZ shift, the response of the median model in Fig. 3a suggests that it is either a weak effect in that model or it is being almost entirely balanced by competing effects. However, there are large differences in aerosol radiative forcing across climate models in both historical and future simulations [86, 87], and it is likely that this forcing uncertainty contributes to the inter-model spread in projected ITCZ shifts.

Although the zonal-mean ITCZ is our focus here, it should be noted that tropical precipitation at individual longitudes is only weakly related to the zonal-mean ITCZ (at least in climate models [21]), and so it is plausible that regional changes in ITCZ location under global warming could be substantially larger than the median predicted zonal-mean change of 0.03°/K. Indeed paleoclimate records suggest the zonal-mean ITCZ has shifted by no more than 1° between present day and either the Last Glacial Maximum or the mid-Holocene [83], yet regional ITCZ migrations of more than 5° have been inferred from paleoclimate reconstructions [83]. A recent extension of the energetic theory for ITCZ location to the zonal direction [22, 74] has potential for advancing understanding and predictions of future changes in regional ITCZ location – we return to this topic in Emerging Theory to Understand ITCZ Responses.

#### Width and Strength

The majority of CMIP5 models (22/32) predict a narrowing of the ITCZ over the twenty-first century [30] (Fig. 3b): The median fractional change in ITCZ width is − 0.52%/K with an interquartile range of 1.17%/K. The fractional ITCZ width changes correspond to a median absolute narrowing of − 0.14°/K, which is associated primarily with a northward shift of the southern ITCZ edge [30] (the observed ITCZ narrowing over recent decades is also due mostly to a northward shift of the southern ITCZ edge [27]). By the small-angle approximation, fractional changes in ITCZ width and area are very similar. ITCZ narrowing contrasts with the overall widening of the Hadley circulation under global warming [39, 41, 42, 88, 89]. The predicted fractional changes in ITCZ width are smaller than fractional changes in the width of the descent region of the Hadley circulation, and the changes are strongly anti-correlated [30]: Climate models that predict a large widening of the dry, subtropical descent region tend to also predict a large narrowing of the wet, tropical ITCZ. Changes in the width of the ITCZ defined using mass versus moisture convergence are well correlated [30], indicating that the predicted narrowing of tropical ascent will coincide with changes in the hydrological cycle near the edges of the ITCZ. The relationship between mass and moisture definitions of the ITCZ suggests that a theory for the mass ITCZ will be useful for interpreting and predicting changes in the moisture ITCZ and in tropical hydroclimates.

To directly compare simulated future changes in ITCZ width to the observed trends shown in Figure S1, we apply the observational analysis technique of Wodzicki and Rapp [27] based on precipitation to the CMIP5 models. Using this technique, we find that models show a narrowing of the Atlantic ITCZ with global warming at a median rate of approximately 3%/K, which is larger than the narrowing of the zonal-mean ITCZ based on the mass streamfunction definition (0.52%/K), but considerably smaller than the observed narrowing over recent decades which we estimated to be 20%/K. Applying this technique used for the observed trends to the Pacific ITCZ, models predict a widening at a median rate of approximately 3%/K, which contrasts with the observed narrowing in the Pacific (Figure S1e). The contrasting responses of the Atlantic and Pacific ITCZs to observed global warming over recent decades versus in model simulations of the future have multiple potentially valid interpretations. One suggests that models are deficient in simulating processes that control ITCZ structure; this is a well known and ongoing issue in climate modeling that often manifests as a “double ITCZ” bias [90, 91]. Another is simply that internal variability, rather than a forced trend, in regional ITCZ width drives the observed variation.

**Fig. 4:**
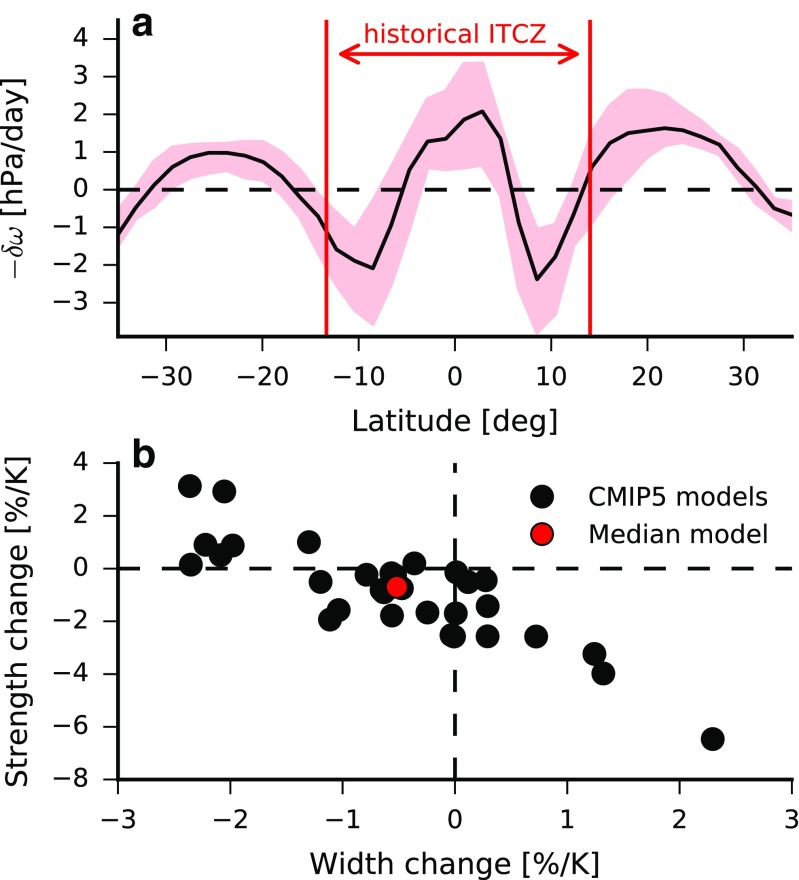
**a** Multimodel-median change at each latitude in (minus) annual- and zonal-mean mid-tropospheric vertical velocity between the historical (1985–2004) and RCP8.5 (2079–2098) simulations (black line). The red shading indicates the interquartile range in vertical velocity changes across models at each latitude. The vertical velocities have been vertically averaged (with mass weighting) from 700hPa to 300hPa. The red vertical lines show the multimodel-median northern and southern edges of the ITCZ in the historical simulations, as defined using the mass streamfunction method. **b** Scatterplot of fractional changes in ITCZ strength versus fractional changes in ITCZ width between the historical and RCP8.5 simulations. The fractional changes in strength and width have been normalized by each model’s global-mean surface-air temperature change. The black dots indicate individual CMIP5 models and the red dot shows the median model changes. The correlation coefficient across models is *r* = − 0.85

Most CMIP5 models (24/32) predict reduced ITCZ strength (i.e., weaker upward motion) under global warming (Fig. 3c), with a median fractional weakening of 0.69%/K and an interquartile range of 1.75%/K. As is the case for changes in ITCZ width, this weakening is more robust across climate models than changes in ITCZ location, though there is considerable inter-model spread. Although the ITCZ weakens with warming on average, this weakening is the small residual between two larger quantities: strongly reduced ascent on the equatorward edges of the ITCZ and increased ascent in the core of the ITCZ [29, 30, 33] (Fig. 4a). This pattern of vertical velocity changes in the ITCZ has been termed the “deep-tropics squeeze” [29] and, in addition to thermodynamically driven changes in atmospheric moisture, it strongly shapes the hydroclimate response to climate change [76, 92]. A weakening of the ITCZ is consistent with the general weakening of the overturning atmospheric circulation with warming [28, 93–97]. In the next section we discuss the relationship between changes in ITCZ width and strength.

## Emerging Theory to Understand ITCZ Responses

### Location

What physical mechanisms drive shifts in the ITCZ? The energetic theory for zonal-mean ITCZ location has been comprehensively reviewed in four recent articles [1, 24–26]. Here, we summarize this body of work, briefly discuss a distinct but complementary dynamical theory for ITCZ location, and outline some outstanding questions.

#### Energetic Theory

The energetic theory for zonal-mean ITCZ location has been developed over the last two decades through a combination of new conceptual insights and a wide array of idealized simulations. This theory relates ITCZ location to interhemispheric contrasts in temperature and net radiative fluxes at TOA [10–21]. The key idea is that warming or cooling of one hemisphere relative to the other necessitates an anomalous cross-equatorial flow of energy into the colder hemisphere and an ITCZ shift.

Using idealized simulations with a “slab” ocean, a collection of studies has advanced our understanding of the zonal-mean ITCZ location by quantitatively connecting interhemispheric contrasts in net TOA radiation, the cross-equatorial energy flux and ITCZ location [11, 13, 14]. Early versions of this energetic theory assumed a passive ocean and a cross-equatorial atmospheric energy flux that is associated entirely with the zonal-mean Hadley circulation, with negligible contributions from transient or stationary eddies. These studies identified the ITCZ location as the latitude where the poleward atmospheric energy flux is zero (the so-called “energy flux equator” [13]). According to this theory, an increase in the interhemispheric TOA radiation contrast (as a result of, say, imposing ice in one hemisphere and increasing its albedo [10]) requires an increase in cross-equatorial energy flux by the Hadley circulation and a shift of the ITCZ further into the hemisphere that is receiving additional radiation.

The relationship between the cross-equatorial energy flux and ITCZ location is central to the energetic theory, as it controls how far the ITCZ must shift in response to interhemispheric radiation asymmetries. Studies suggest that the ITCZ moves 3° latitude per 1 petawatt (PW) of cross-equatorial atmospheric energy flux [17, 26, 98]. Using this diagnosed relationship, Donohoe and Voigt [26] argue that because interhemispheric contrasts in radiative forcings and feedbacks are expected to be substantially smaller than 1 PW under global warming, future changes in ITCZ location will be less than 1° latitude, consistent with our analysis of projected (Fig. 3a) and observed ITCZ changes (Figure S1a,d). However, simulations with prescribed glacial and interglacial boundary conditions find that the 3° latitude per 1 PW cross-equatorial flux relationship for ITCZ location does not always hold [21].

Building on the work of Kang et al. (2008, 2009) [13, 14], a linearization of the atmospheric energy budget suggests that the slope of the quasi-linear relationship between ITCZ location and cross-equatorial energy flux is determined by the net energy input to the tropical atmosphere [19, 25], which itself is modulated by cloud processes, radiative transfer in the atmosphere, and ocean heat uptake:

5 $$ \phi_{\text{ITCZ}} \approx - \frac{1}{a} \frac{F_{0}}{S_{0} - L_{0} - O_{0}},  $$

where *ϕ*_ITCZ_ is identified here as the latitude of the energy flux equator, *F* is the vertically integrated meridional moist static energy flux by the atmosphere, *S* is the shortwave atmospheric heating, *L* is the longwave atmospheric cooling, and *O* is the ocean heat uptake. The subscripts (⋅)_0_ indicate that the quantities are evaluated at the geographical equator. Net energy input to the tropical atmosphere (*S*_0_ − *L*_0_ − *O*_0_) is expected to increase under global warming [30] and this would, according to Eq. 5, weaken the ITCZ location–energy flux relationship and shift the ITCZ towards the equator without necessitating a change in the cross-equatorial energy flux, *F*_0_. Thus, a change in net energy input is a possible mechanism for shifting the ITCZ under global warming, and likely contributes to the inter-model spread in predicted ITCZ shifts (Fig. 3a).

The energetic theory for ITCZ location was developed largely under the assumption of a passive ocean. However, new research [1, 99–103] has demonstrated that the inclusion of a dynamic ocean strongly damps ITCZ shifts (by a factor of approximately three according to one estimate [102]) and reduces the sensitivity of ITCZ location to interhemispheric radiation contrasts. This damping of ITCZ migrations by the ocean is consistent with the muted observed and projected ITCZ location shifts discussed above. (For a detailed discussion of the role of ocean coupling in ITCZ dynamics, see the recent review by Kang et al. [1].)

#### Dynamical Theory

An alternative dynamical theory for ITCZ location, less prominent in the literature than the energetic theory, is based on principles of tropical atmospheric dynamics [104–107]. Assuming convection is sufficiently active in the ITCZ such that the lapse rate is close to moist adiabatic [108] and further assuming the Hadley circulation conserves angular momentum in the free troposphere, the ITCZ location is expected to lie just equatorward of the maximum in boundary-layer moist static energy. Over oceans, assuming near-surface relative humidity is sufficiently constant in space, the dynamical theory implies that the ITCZ location is just equatorward of the maximum in sea-surface temperature (SST). The dynamical theory is broadly verified by an observational analysis of monsoons though the presence of dry, shallow circulations can complicate the picture [109]. Although this dynamical theory is diagnostic in the sense that the ITCZ location can be determined only if the distribution of boundary-layer moist static energy is known, it nevertheless provides a distinct framework to the energetic theory with which to understand ITCZ migrations. (See Shekhar & Boos [110] for a systematic comparison of the energetic and dynamical theories for ITCZ location.)

#### Outstanding Questions

The energetic theory, summarized by Eq. 5, represents substantial progress in our understanding of the zonal-mean ITCZ location. A priority for future work should be to use this theory (and extensions to it) to identify the processes driving the inter-model uncertainty in ITCZ shifts (Fig. 3a). There are a number of candidate processes, including model differences in interhemispheric contrasts in the radiative forcings and feedbacks that determine *F*_0_, differences in net energy input to the tropical atmosphere (*S*_0_ − *L*_0_ − *O*_0_) and differences in the partitioning of a given cross-equatorial energy flux between the atmosphere and ocean. These processes are represented in climate models by a wide variety of components including those simulating cloud physics, radiative transfer, ocean dynamics and ice sheets. Only after identifying the dominant contributors to the spread in projected ITCZ shifts can reducing this uncertainty become feasible.

On the theoretical side, there are opportunities to further develop our physical interpretation of the processes controlling ITCZ location and build towards a complete, predictive theory. The energetic theory for ITCZ location assumes the cross-equatorial atmospheric energy flux is due entirely to the zonal-mean Hadley circulation and that energy transports by transient and stationary eddies are negligible. However, transient and stationary eddies are not generally negligible in the energy and water budgets of the tropical atmosphere in both the observed climate and in models [18, 21, 30, 36, 111–114]. As a first step towards incorporating the influence of eddies into the energetic theory, the total cross-equatorial energy flux *F*_0_ could be decomposed into mean (Hadley circulation), transient-eddy and stationary-eddy components (*F*_0_ = *F*_mean_ + *F*_transient_ + *F*_stationary_) and the relative influences of these processes on the climatological ITCZ and on ITCZ shifts could then be diagnosed. Such an analysis would inform as to whether a detailed knowledge of eddies at low latitudes is necessary for an understanding of ITCZ location.

The zonal-mean theory for ITCZ location undoubtedly gives insights into why the ITCZ lies north of the equator in today’s climate, and provides a quantitative framework with which to examine past and future ITCZ shifts. However, the impacts of climate change are experienced regionally and models suggest that local tropical precipitation changes are not tightly coupled to the zonal-mean ITCZ location [21]. Furthermore, if we seek to develop a robust understanding of tropical circulation and precipitation in past climates, we need to advance the theory for ITCZ location beyond the zonal mean and make hindcasts that can be compared to paleoclimate data which are inherently regional. Recently, an important step towards a regional theory for ITCZ location has been made by generalizing the energetic theory through the definition of an “energy flux potential” [22, 74] which essentially extends the theory to the zonal direction. Thus far, this extended theory has been applied to explain continental rainfall shifts in the mid-Holocene [22] and the observed seasonal and interannual behavior of the zonally anomalous ITCZ over recent decades [74]. The response of regional tropical precipitation to climate change is highly uncertain [96, 115], and this new method should now be applied to identify the processes contributing to inter-model uncertainty on regional scales.

### Width and Strength

In comparison to the ITCZ location, we have only a limited mechanistic framework for interpreting changes in the width and strength of the ITCZ. Arguably these features are as important to understand and predict as ITCZ location, given that changes in ITCZ width and strength are likely to have important implications for hydroclimates in tropical regions.

Here, we review the current state of knowledge on ITCZ width and strength, demonstrate that changes in these quantities are strongly anti-correlated across CMIP5 models, and outline our vision for how progress can be made to advance understanding of these key features of the ITCZ structure.

#### Controls on ITCZ Width

The ITCZ is narrower than the neighboring subsiding regions of the Hadley circulation [36] and is a region of widespread moist convection [116]. Consequently, theories for the area fraction of moist convection have potential relevance for the ITCZ width. Bjerknes (1938) [117] used a thermodynamic argument based on dry and saturated moist adiabatic lapse rates to argue that moist convection tends to occupy an updraft region that is narrow relative to the downdraft region, although he neglected temperature tendencies due to radiative and surface fluxes. Later, analytical and idealized-modeling studies of the Walker circulation found that the area of ascending motion depends upon the SST gradient, the gross moist stability,^2^ cloud-radiative feedbacks and atmosphere-ocean coupling [34, 119]. Following these studies that considered the convective area fraction quite generally, various idealized-modeling studies noted a dependence of ITCZ width on the dynamical core and model resolution [120], the convective parameterization [14], the strength of horizontal diffusion of moisture [35], the radiative effects of clouds and water vapor [38, 121], and the longwave optical thickness [36]. It is clear from these idealized models, and indeed from observations [27] and comprehensive models [29, 30], that the ITCZ width is influenced by a variety of climate processes.

Examination of the narrowing of the ITCZ in response to global warming has stimulated further interest in the physics governing ITCZ width. A number of mechanisms have been put forward to explain this narrowing. The “upped-ante” hypothesis based on analyses of the atmospheric moisture and moist static energy budgets [31–33] argues that decreases in precipitation on the margins of tropical convective regions under global warming are caused by boundary-layer advection of anomalously dry air from neighboring non-convective regions. This advective drying in the boundary layer, combined with enhanced upper-tropospheric warming, inhibits convection on the ITCZ edges and reduces precipitation. However, the upped-ante hypothesis is not a complete explanation; it does not account for processes such as the divergence of moist static energy out of the tropics by transient eddies and changes in net energy input to the atmosphere, which have been shown to affect ITCZ width in a warming climate [30].

An alternative hypothesis using two heuristic models linking moisture, vertical velocity, and rainfall distributions suggests that an increase in the skewness of the vertical velocity distribution under global warming could explain ITCZ narrowing [37]. This increase in skewness arises naturally from the asymmetric effect of latent heating on vertical motions, consistent with earlier work [117]. However, the extent to which this conceptual model can explain the observed and projected ITCZ narrowing in more comprehensive models has yet to be fully explored.

Recently, a new diagnostic equation linking the ITCZ width to energy transports in the climate system has been derived [36] and applied to quantify the processes contributing to ITCZ narrowing under global warming [30] (see Section 3 of Byrne and Schneider [36] for a derivation and discussion of this equation). By combining the mass and moist static energy budgets of the Hadley circulation, the equation demonstrates that the ITCZ narrows or widens due to changes in four processes: (i) gross moist stability, (ii) net energy input to the atmosphere [the *S* − *L* − *O* term from Eq. 5], (iii) advection of moist static energy by the Hadley circulation, and (iv) divergence of moist static energy from the tropics by transient eddies. Interestingly, because the Hadley circulation mass budget links area fractions and vertical velocities in the ITCZ and descent regions, processes in both the ITCZ and descent regions can change the ITCZ width (though local processes within the ITCZ are typically dominant because the gross moist stability is small there [30]).

The theory described above quantifies how changes in four distinct climate processes influence ITCZ width, and the physical interpretation for these influences is straightforward. Take net energy input to the atmosphere as an example. If climate is perturbed such that net energy input to the ITCZ increases, perhaps due to increased CO_2_ concentrations and reduced longwave cooling, the ITCZ circulation must strengthen to transport this excess energy poleward (assuming a thermally direct mean divergent circulation, i.e., that gross moist stability is positive). All else being equal in this thought experiment, the vertical mass flux in the descent region of the Hadley circulation remains unchanged, implying that the ITCZ narrows so as to maintain equal and opposite mass fluxes in the ITCZ and descent region (Figure S2, see Supplementary Material). Analogous physical arguments can be made to understand how the other three terms in the diagnostic equation [36] impact ITCZ width.

In CMIP5 simulations, ITCZ narrowing has been found to be driven by steepening of the meridional moist static energy gradient with global warming—this enhances cooling of the ITCZ by Hadley circulation advection and transient-eddy divergence, which affect the tropical vertical velocity and hence the ITCZ width [30]. Energy-transporting transient eddies originating in mid-latitudes are thus an important non-local influence on the ITCZ width. Over oceans, moist static energy is strongly controlled by SST, which itself is shaped by atmosphere-ocean coupling processes. As a result, a change in SST pattern—and in particular the meridional SST gradient—is a means by which the ocean can influence the ITCZ width (in addition to the ocean’s central role in setting net energy input to the atmosphere via surface fluxes). Inter-model spread in the response of the tropical circulation to climate change has been linked to differences in SST patterns [122, 123], and the diagnostic equation described above gives new physical insights into why the ITCZ depends on SST patterns. ITCZ narrowing due to steepening meridional moist static energy gradients is opposed by a widening tendency due to changes in gross moist stability and increases in shortwave absorption in the atmosphere resulting from cloud and clear-sky effects [30]. The processes that narrow the ITCZ with warming overcome the widening processes in most, but not all, CMIP5 models (Fig. 3b). Clearly, projected changes in ITCZ width result from a delicate balance between competing effects, and examining in more detail how well climate models capture this balance is a challenge for future work.

The processes that this diagnostic equation identifies as influencing ITCZ width [36]—including gross moist stability, cloud feedbacks, energy/moisture advection, and moisture transport by transient eddies—were identified previously as being important for convective area in the tropics [32–35, 119]. The breakthrough in these more recent studies [30, 36] has been to derive a quantitative relationship that permits an evaluation of the relative importance of the difference processes for changes in ITCZ width across models and climates.

#### Relationship Between ITCZ Width and Strength

Changes in ITCZ width and strength under global warming are strongly anti-correlated across CMIP5 models (Fig. 4b). Why does this relationship between width and strength exist? The rate of total mass transport within the ITCZ is given by Ψ_ITCZ_ = −*A*_ITCZ_*ω*_ITCZ_/*g*. Under global warming, the total atmospheric mass transport is expected to weaken [96, 97, 124], consistent with the imbalance between rapid increases in atmospheric water vapor versus slower increases in global precipitation [94] and with differential increases in static stability and radiative cooling in descending regions [93]. In the ITCZ, weaker vertical mass transport can be accomplished by a narrowing of the ascent region (*δA*_ITCZ_/*A*_ITCZ_ ≈ *δW*_ITCZ_/*W*_ITCZ_ < 0), a weakening of the circulation (*δω*_ITCZ_/*ω*_ITCZ_ < 0), or a combination of both effects. Linearizing the definition of Ψ_ITCZ_ above, it is straightforward to show that a given fractional change in vertical mass transport approximately constrains the sum of fractional changes in ITCZ width and strength:

6 $$ \frac{\delta {\Psi}_{\text{ITCZ}}}{{\Psi}_{\text{ITCZ}}} \approx \frac{\delta W_{\text{ITCZ}}}{W_{\text{ITCZ}}} + \frac{\delta \omega_{\text{ITCZ}}}{\omega_{\text{ITCZ}}}.  $$

Equation 6 illustrates, for example, that in a given model a large fractional decrease in ITCZ width must be compensated for by a small fractional decrease in ITCZ strength in order for the fractional change in mass transport to equal the value determined by the separate processes mentioned above. This compensation is consistent with the anti-correlation found across CMIP5 models (Fig. 4b). In the majority of these models, both ITCZ narrowing and weakening contribute to the reduced mass transport under global warming (Fig. 4b).

#### Path Forward

Observed and projected changes in ITCZ width and strength are more robust than changes in ITCZ location—a clear target for future research is to understand these changes. The development of the energetic theory for ITCZ location has come about through a combination of theory and a hierarchy of simulations. We believe that this hierarchical approach [125, 126] should be the template for new research to develop, test and refine hypotheses for how the ITCZ width and strength respond to climate change.

A starting point on this hierarchy of simulations would be to use a dry dynamical model in the Held-Suarez configuration [127] with localized thermal forcings systematically applied at different latitudes and pressure levels. This approach would construct response functions for ITCZ width and strength analogous to those being used to understand the sensitivities of mid-latitude dynamics and the Hadley circulation to internal climate variability and external forcings [128–130]. Response functions would show the broad range of possible ITCZ width and strength responses in the absence of processes such as latent heating and radiative transfer which play important but complicating roles in ITCZ dynamics in comprehensive models and in Earth’s climate. Moving up the model hierarchy, moist aquaplanet models with simplified gray radiation schemes are ideal tools with which to assess how latent heating and surface fluxes couple to the ITCZ, and have been used extensively in tropical atmospheric dynamics research [14, 19, 36, 131, 132]. Moist aquaplanets with prescribed surface, atmospheric, and TOA thermal forcings—analogous to those described above for a dry model—would establish a basic understanding for whether ITCZ width and strength are more sensitive to local or extratropical forcing, or to heating in the lower or upper troposphere. Aquaplanet simulations using the “radiation-locking” technique [121, 133] could further be used to decompose how radiative effects associated with CO_2_, aerosols, water vapor and clouds control the ITCZ response to climate change. The ITCZ is a region of frequent moist convection and a computational hierarchy to understand ITCZ sensitivity should include a model with the capability to explicitly simulate deep convection. Limited-domain, fixed-SST, convection-permitting simulations are becoming increasingly feasible, and novel “aquachannel” configurations have recently been used to study the climatological ITCZ [134]. Performing analogous convection-permitting simulations with a range of SSTs would be a first step towards accounting for the role of explicit atmospheric convection in modulating the ITCZ response to climate change.

Ultimately, our aims should be to advance fundamental understanding of the ITCZ width and strength and to predict their sensitivities to imposed forcings, and these aims require an overarching conceptual model or theory. The energetic theory of Byrne and Schneider [36] is quantitative but diagnostic; it does not permit *a priori* predictions for changes in ITCZ width given, say, the SST change (additional theories for the responses of gross moist stability, clouds, mid-latitude eddies and other processes would also be required). Theoretical progress on ITCZ width could be made by adapting theory linking tropical circulations to boundary-layer moist static energy and SSTs [105–108] in order to derive dynamical constraints for the latitudes of the ITCZ boundaries. Given the tight coupling between changes in ITCZ width and strength (Fig. 4b), a predictive theory for either ITCZ width or strength would allow us to infer changes in both quantities.

#### Outstanding Questions

We now list specific questions related to ITCZ width and strength on which we believe progress can readily be made. It is established that the mean tropical circulation weakens under global warming, but why does this weakening manifest as a “deep-tropics squeeze” within the ITCZ (Fig. 4a)? This pattern of circulation change will undoubtedly shape future hydroclimate in the tropics, and could influence global radiation balance and climate sensitivity via its impact on the area of convective anvil clouds [3]. Analyses of atmospheric moisture and moist static energy budgets [33], and equatorial SST warming patterns [135, 136], suggest that gross moist stability influences the pattern of vertical velocity (and hence precipitation) changes in the ITCZ. However, a simple thermodynamic scaling for gross moist stability [137] only partly captures its response to external forcing in climate models [138]. As a result, constructing a predictive theory for tropical vertical velocity and ITCZ strength using atmospheric moisture and energy budgets remains a challenge. The horizontal momentum budget and first-baroclinic mode of the tropical atmosphere have been used to construct a quantitative theory for vertical velocity in subtropical stationary circulations [139], and such an approach could be adapted to the ITCZ problem to better understand the “deep-tropics squeeze”.

Most models predict a narrowing and weakening of the ITCZ under global warming, but do models in turn simulate a widening and strengthening of the ITCZ in cold climates of the past, such as the Last Glacial Maximum? If so, this may have implications for interpreting tropical paleoclimate data. The responses of temperature [140], humidity [141], the water cycle [92] and atmospheric circulations [97] to climate change differ strongly over land and ocean—is this the case for the ITCZ width and strength? This should be evaluated, and if so, developing new theories and conceptual models to capture these land-ocean ITCZ contrasts should be a priority. Finally, in recent years substantial progress has been made in understanding the role of the ocean in controlling ITCZ location shifts [1, 99–103] but how atmosphere-ocean coupling modulates changes in ITCZ width and strength is largely unknown. A first step towards quantifying the importance of ocean coupling would be to remove the thermodynamic coupling by analyzing fixed-SST simulations and comparing the ITCZ width and strength changes under prescribed global warming to the responses in fully coupled simulations.

## Discussion

The ITCZ provides water to billions of people in tropical regions and any responses of the ITCZ to climate change need to be understood and accurately predicted. Large shifts in ITCZ location over Earth’s history have been recorded in paleoclimate data, but in recent decades it has been other aspects of the ITCZ structure that have been changing in response to climate change. Over the Atlantic and Pacific oceans the ITCZ has narrowed and its precipitation rate has intensified but the location has remained nearly unchanged. These observed trends are qualitatively consistent with projections for the ITCZ: Models simulate narrowing and weakening of the ITCZ circulation in a warming climate, but no robust change in location. Interestingly, we show that changes in ITCZ width and strength are strongly anti-correlated across models; this relationship can be understood by considering constraints on the atmospheric vertical mass transport. Uncertainties in the responses of the ITCZ to climate change are substantial, and reducing these uncertainties is contingent upon improving our fundamental understanding of ITCZ dynamics, and in particular the processes shaping ITCZ width and strength. The development of new conceptual insights into ITCZ dynamics will help targeted improvement of climate models and their simulation of the ITCZ, and may offer opportunities to develop “emergent constraints” to narrow uncertainty in existing CMIP5 simulations and in the impending CMIP6 simulations [142]. (An emergent constraint on the response of tropical high-altitude cloud fraction to global warming has recently been proposed [4], but whether this informs the ITCZ width response is an open question.)

Climate dynamicists have been successful in developing an energetic theory that relates ITCZ location to cross-equatorial energy transport. Extensions to this theory have incorporated atmosphere-ocean coupling and energy input to the tropical atmosphere, and the theory has recently been generalized so as to be applicable beyond the zonal mean. An opportunity for further development is to account for the roles of atmospheric transient and stationary eddies in setting the modern-day ITCZ location and in modulating its response to climate change. A distinct but complementary dynamical theory for ITCZ location has also been developed. These advances in understanding ITCZ location were partly motivated by the paleoclimate record, and were driven by key conceptual insights combined with idealized simulations focused on physical mechanisms.

Theories for the ITCZ width and strength are neither as developed nor as extensively tested as those for ITCZ location, but are beginning to emerge. A study with two heuristic models of precipitation indicates a decrease in the rain area fraction as the climate warms, and the upped-ante hypothesis predicts reductions in precipitation on the margins of convergence zones; both are consistent with observed and predicted ITCZ narrowing. An energetic theory, analogous to that developed for ITCZ location, quantifies how processes including gross moist stability and net energy input to the atmosphere contribute to changes in ITCZ width, and this theory has been applied to projected ITCZ changes. This energetic theory is diagnostic rather than predictive, but it suggests that changes in ITCZ width are related to the climatological gross moist stability; gross moist stability is thus a potential emergent constraint on ITCZ width. Although it is well established that the total atmospheric mass transport weakens in a warming world, resulting in the anti-correlation between changes in ITCZ width and strength across climate models, we do not yet have a complete theory to capture the “deep-tropics squeeze” pattern of these circulation changes.

In this review, we have focused on the annual- and zonal-mean ITCZ structure, which is a sensible starting point for interpreting the real-world tropical circulation. However, a goal for climate dynamics is to predict regional responses to climate change [143] and ultimately our challenge is to develop theories for the ITCZ that apply in different seasons and regions. Recent work has extended the energetic theory for ITCZ location to the zonal direction and similar advances in theories for ITCZ width and strength now need to be made. Motivated by the development of our knowledge of ITCZ location through conceptual insights and the use of idealized models, we propose using a hierarchy of simulations to broadly explore the processes determining ITCZ structure and to stimulate further mechanistic understanding. Such an approach is now needed in order to reduce the considerable uncertainties in how the ITCZ will change in the future.

## Electronic supplementary material

Below is the link to the electronic supplementary material.

(PDF 131 KB)

## Footnotes

1 The models analyzed here are ACCESS1-0, ACCESS1-3, BCC-CSM1-1, BCC-CSM1-1-M, BNU-ESM, CESM1-BGC, CESM1-CAM5, CMCC-CESM, CMCC-CM, CMCC-CMS, CNRM-CM5, CSIRO-Mk3-6-0, CanESM2, EC-EARTH, FGOALS-g2, FIO-ESM, GFDL-CM3, GFDL-ESM2G, GFDL-ESM2M, HadGEM2- AO, HadGEM2-ES, INMCM4, IPSL-CM5A-MR, IPSL-CM5B-LR, MIROC-ESM, MIROC-ESM-CHEM, MIROC5, MPI-ESM-LR, MPI-ESM-MR, MRI-CGCM3, NorESM1-M, and NorESM1-ME.

2 The gross moist stability is a bulk measure of moist static energy stratification that is frequently used in analyses of tropical atmospheric dynamics [14, 118].
