# Supplementary material for: Response of the Intertropical Convergence Zone to Climate Change: Location, Width, and Strength
Source: Curr Clim Change Rep. 2018 Aug 9;4(4):355–70. doi: 10.1007/s40641-018-0110-5 (PMC6411165; doi:10.1007/s40641-018-0110-5)
Supplement: Supplementary file 1 — (PDF 131 KB) [file 40641_2018_110_MOESM1_ESM.pdf]

Response of the Intertropical Convergence Zone to Climate Change: Location, Width and Strength

Supplementary Material

Michael P. Byrne, Angeline G. Pendergrass, Anita D. Rapp & Kyle R. Wodzicki

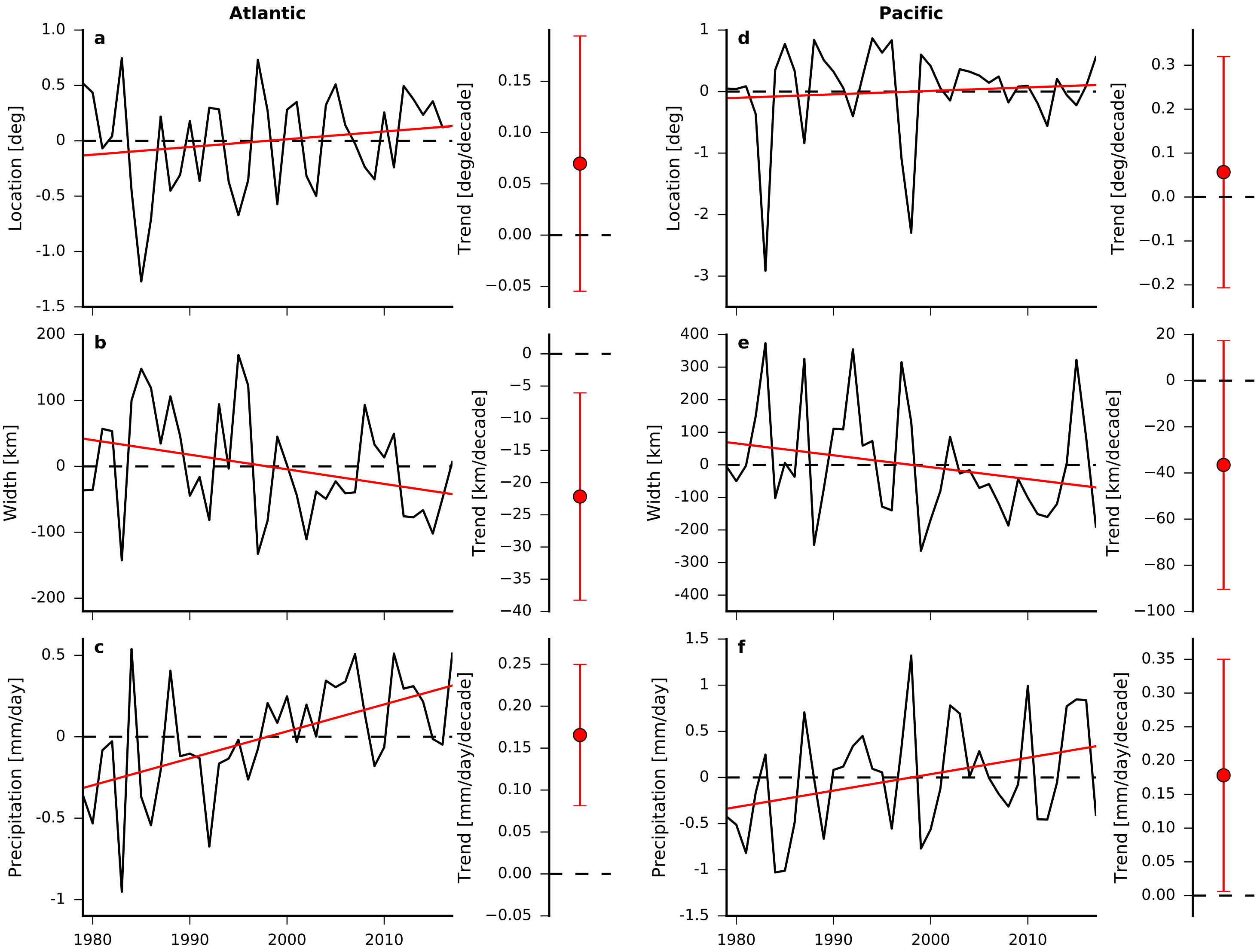

Figure S1: Timeseries of anomalies in annual-mean Atlantic basin ITCZ (15°W–45°W): (a) location from ERA Interim, (b) width from GPCP and (c) precipitation intensity from GPCP for 1979–2017. Panels (d)–(f) show the same quantities but for the Pacific basin ITCZ (160°E–100°W). The best-fit trend for each timeseries is also plotted (red lines and dots). The error bars on the trends show the 90% confidence intervals corrected to account for autocorrelation in the timeseries following the method of Leith (1973) [1].

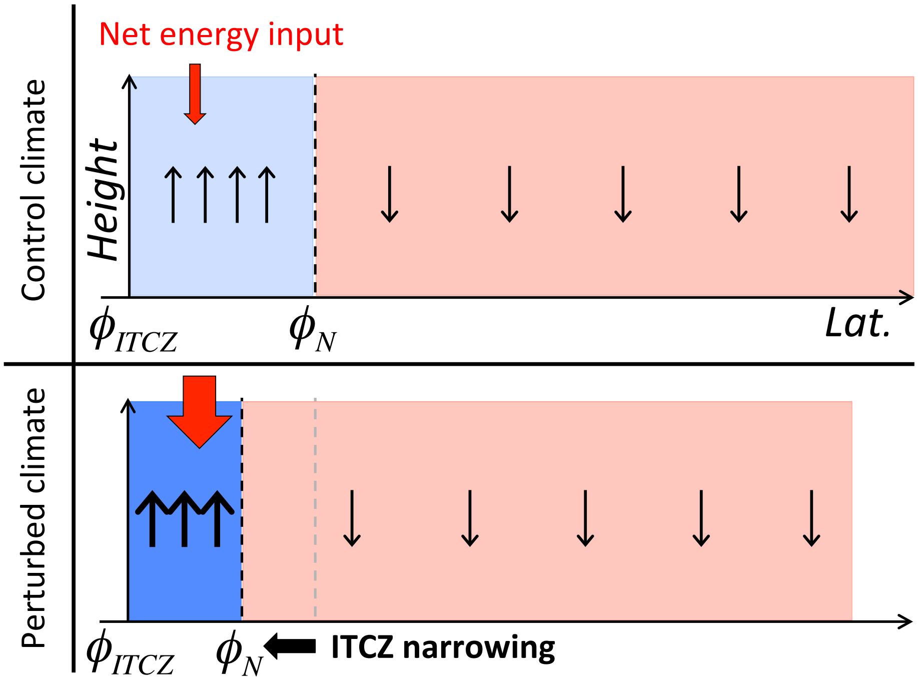

Figure S2: Schematic of the northern cell of the Hadley circulation depicting how increases in net energy input tend to narrow the ITCZ. The blue shading indicates the ITCZ and the red shading indicates the descent region; the darker the shading, the larger the magnitude of the vertical velocity. Adapted from Byrne & Schneider (2016) [2].

References

[1] C.E. Leith, The standard error of time-average estimates of climatic means, J. Appl. Meteor. **12**, 1066 (1973)

[2] M.P. Byrne, T. Schneider, Energetic constraints on the width of the intertropical convergence zone, J. Climate **29**, 4709 (2016)
